# From grassroots to strategy: advancing laboratory sustainability at Utrecht university

**DOI:** 10.1039/d6ra04362c

**Published:** 2026-06-02

**Authors:** Greta Di Marco, Elise J. Heesbeen, Boaz Chemtob, Jurriaan W. Reiprich, Katerina A. T. Xenaki, Mies J. van Steenbergen, Antoinette van den Dikkenberg, Wim van de Leeuw, Anne F. Nelissen, Joep Sprangers, Bianca Schell, Rosalinde Masereeuw, Jesus Rosales Carreon, Tina Vermonden

**Affiliations:** a Department of Pharmaceutical Sciences, Faculty of Science, Utrecht University Utrecht 3584 CG the Netherlands e.j.heesbeen@uu.nl; b Operational Management, Health, Safety and Environment, Faculty of Geosciences, Utrecht University Utrecht 3584 CB the Netherlands; c Faculty Office, Faculty of Veterinary Medicine, Utrecht University Utrecht 3584 CL the Netherlands; d Operational Management, Research and Valorisation Policy, Faculty of Science, Utrecht University Utrecht 3584 CD the Netherlands; e Center for Molecular Medicine, University Medical Center Utrecht, Universiteitsweg 100 Utrecht 3584 CX the Netherlands; f Department of Chemistry and Centre for Synthetic Biology, Technical University of Darmstadt Peter-Grünberg-Straße 4 64287 Darmstadt Germany; g Department of Physics, University of Konstanz, Universitätsstraße 10 78464 Konstanz Germany; h Copernicus Institute of Sustainable Development, Faculty of Geosciences Utrecht 3584 CB the Netherlands

## Abstract

Scientific research, especially in experimental disciplines, depends on laboratories (labs), which are among the most energy- and resource-intensive facilities within universities. Despite their significant environmental footprint, labs are often overlooked in sustainability strategies, even though their contribution is crucial to achieving institutional and global climate goals. This Utrecht university case study presents the development of Green Labs initiatives and the use of the Laboratory Efficiency Assessment Framework (LEAF) as a scalable approach towards structured sustainability practices. We conducted a partial assessment of the university-wide lab-related environmental impact and a detailed evaluation of environmental and social outcomes from LEAF-bronze (entry-level) implementation in the Department of Pharmaceutical Sciences. Results show that LEAF low-cost measures produced measurable environmental benefits while enhancing user motivation and satisfaction. Post-implementation monitoring of a 910 m^2^ lab-facility showed a 5% reduction in total electricity use and a 38% decrease in targeted equipment electricity use, saving 15 MWh, 2.9 t CO_2_e, and 1500 EUR annually. Waste segregation improved substantially, particularly in plastic and paper recycling, while hazardous waste volumes remained stable. Surveys of lab users (*n* = 99) and LEAF-team members (*n* = 18) indicated high satisfaction, increased motivation for sustainable practices, and perceived -benefits for lab organization, safety, and team cohesion. Beyond technical interventions, the findings underscore the importance of grassroots initiatives, community engagement, and institutional support in driving systemic, long-lasting change. This case study offers a replicable model for embedding sustainability in academic labs and advancing the transition toward climate-neutral, resource-efficient, and socially responsible research environments.

## Abstract 2

This case study offers a replicable model for embedding sustainability in academic labs and advancing the transition toward climate-neutral, resource-efficient, and socially responsible research environments.

## Graphical abstract

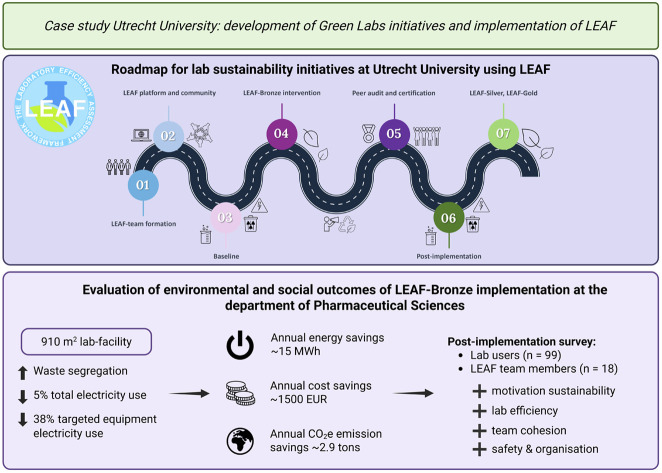

## 1. Introduction

As the climate and ecological crises intensify, driven by human activities that exceed the planet's regenerative capacity, society faces an urgent need to achieve climate neutrality and adopt more sustainable resource use.^1–3^ Academic and scientific institutions are central to this effort, both in advancing understanding of global challenges and in developing solutions. In recent decades, these institutions have increasingly integrated environmental sustainability into their strategic plans, including policies on energy-efficient buildings, catering and transportation.^4^ Yet, the environmental impact of research itself has remained largely overlooked, with practices that remain resource-intensive and unsustainable.^5–8^

A turning point occurred when researchers began scrutinizing laboratory (lab) operations, which emerged as disproportionately carbon-intensive compared to other organizational sectors.^6,7^ Labs are inherently complex environments, varying in function and activities across research, education, diagnostics, and clinics, and are typically embedded within broader institutional structures. They are generally classified as wet or dry: wet labs, designed for handling chemical, biological, or clinical materials, require strict safety controls and specialized training to prevent spills and contamination, whereas dry (or computational) labs involve minimal hazardous materials but may contain experimental equipment and large data storage infrastructure.^9^ Labs are among the most resource-intensive facilities at universities, with energy use and hazardous waste generation being up to ten and five times higher per square meter, respectively, than standard office spaces.^10–12^ Their reliance on resource-intensive supply chains, particularly single-use plastics and hazardous substances, further amplifies their footprint.^7,13^ Indeed, procurement of lab materials constitutes the largest contributor to labs' overall carbon emissions and biodiversity footprint.^8,14^ Inefficiently designed or duplicated experiments, exacerbate these impacts.^15^

The Green Labs movement emerged in response to growing awareness of the substantial environmental footprint of scientific research. Initially driven by individual researchers through grassroots initiatives to reduce waste and energy use within their own workspace,^6,16^ these efforts gradually evolved into more organized communities, including international organizations such as the US-based NGO My Green Lab, the British program LEAF (Laboratory Efficiency Assessment Framework) and the Sustainable European Laboratories (SELs) network. An increasing number of institutions worldwide have started adopting sustainable lab practices, thereby reducing resource consumption, lowering carbon footprints, and achieving cost savings.^7,17,18^ Energy savings of 20–50% in targeted areas are typically realized by improving equipment efficiency, such as optimizing ventilation and cold storage systems.^7^ Waste reduction is supported by circular economy principles, adherence to the ‘reduce, reuse, recycle’ hierarchy, and shared use of resources and equipment.^7,11,13,19,20^ Sustainable procurement is facilitated by sustainability labels, which provide clear information on product environmental impact.^21^

To facilitate the sustainability transition in research environments, various guidelines and certification schemes have been established to support and incentivize sustainable practices.^22,23^ Among these, LEAF and My Green Lab® certification are the most widely adopted, targeting energy, water use, waste, procurement, and sample management while promoting behavioral change across lab operations.^23^ More recently, dry labs have also gained attention for their high energy demands, leading to the development of dedicated tools such as the open-access Green DiSC certification.^24,25^ Lab sustainability frameworks introduce standardized procedures that enhance operational efficiency while fostering environmental awareness and engagement among researchers.^23^ Certification schemes accelerate adoption, improve institutional visibility, and support compliance with emerging sustainability-focused funding and publication requirements.^26,27^

Despite the global expansion of the Green Labs movement, sustainable lab practices have not yet become standard across the scientific community, concentrated in select countries or institutions and remaining uneven among them.^23,28^ Broader adoption is hindered by limited awareness, misconceptions about complexity and costs, and barriers such as institutional culture and funding. Systemic, lasting change requires strong institutional support, complemented by continued outreach, targeted incentives, and engagement from key stakeholders, including funders, publishers, industry, and policymakers.^28^

In its strategic plan, Utrecht university identifies sustainability as a core pillar, with 2030 targets including carbon neutrality, circular resource use, and enhanced biodiversity.^29^ To advance these goals, facility management (which includes the sustainability office) oversees sustainability programs on infrastructure, energy, waste and procurement, but labs were initially overlooked due to limited awareness of their specific footprint and operational complexity. In response, bottom–up initiatives have emerged through faculty-based Green Teams, grassroots communities of staff and students promoting sustainability in their environments with activities shaped by member expertise and interests.^16,30^ For lab operations, their efforts have focused mainly on LEAF certification, resulting in an engagement rate of 33% among research labs located in university buildings. Of these, 8% have achieved bronze (starting level) certification and 1% silver (middle level). Building on this progress, a university-wide Green Labs Task Force of over 35 stakeholders, including Green Team members, operational services, and the sustainability office, was recently established to systematically embed sustainability across lab operations by aligning initiatives, integrating bottom–up and top–down approaches, and fostering community building.

This study aimed to examine the impact of lab sustainability implementations at Utrecht university and highlight the university's experience in scaling these practices across all labs, including achievements and ongoing challenges. Therefore, we conducted a partial assessment of the university-wide lab-related environmental impacts (Section 2.1), focusing on waste generation, energy use, and procurement, and a detailed evaluation of environmental and social outcomes from LEAF-bronze (entry-level) implementation in the Department of Pharmaceutical Sciences (Section 2.2). Finally, Section 2.3 outlines strategies and recommendations to further expand lab sustainability efforts, aiming to maximize effectiveness and impact. By showcasing Utrecht university's experience, this study aims to drive the adoption of sustainable lab practices and provide unique combined environmental and social evidence of lab sustainability implementation and institutionalization, offering a model for other universities.

## 2 Experimental

### 2.1 Methodology for the assessment of LEAF-bronze (results in Section 2.2)

#### 2.1.1 Case study selection

This case study was selected because Utrecht university represents a mature, institution wide example of lab sustainability implementation, meeting our criteria of (i) demonstrated progress in reducing environmental impacts across waste, energy, and procurement; (ii) documented experience with LEAF-bronze adoption at the departmental level; and (iii) active efforts to scale and institutionalize sustainable practices, enabling an in depth assessment of both environmental and social outcomes relevant to other universities.

#### 2.1.2 Study setting for LEAF-bronze implementation

Within Utrecht university's Faculty of Science, the Department of Pharmaceutical Sciences comprises five research divisions, four having wet labs including pharmaceutics (Px) and pharmacology (Pcol), and coordinates bachelor's and master's programs involving student lab work. Its research integrates biological and chemical methods, making it representative of Life Sciences labs. Section 2.2 reports the main findings on environmental (Px only) and social outcomes (Px and PCol) of lab sustainability intervention, specifically following LEAF-bronze criteria. Px and PCol divisions comprise 16 and 18 lab rooms, with total areas of 910 and 785 m^2^ and approximately 60 and 80 regular lab users, respectively. The environmental outcomes were assessed by monitoring electricity and waste output before and after LEAF implementations. The social outcomes were assessed after LEAF intervention *via* a general survey of all divisional members and a targeted survey of LEAF-team participants directly involved in the implementations.

#### 2.1.3 Monitoring phases for environmental outcomes

For Px Labs, the baseline and post-implementation phases each spanned 12 weeks and were conducted one year apart to ensure seasonal comparability and controlled external factors. The baseline phase occurred from 5 February 2024 to 28 April 2024, and the post-implementation phase from 3 February 2025 to 26 April 2025.

#### 2.1.4 Energy consumption of equipment (excluding fume hoods)

Excluding fume hoods, equipment energy consumption was measured using plug-in energy meters (Ecosavers). Annual energy consumption was calculated using eqn (1):

1 *E*_annual_ = *E*_hour_ × *t*_annual_

where *E*_annual_ is the annual electricity consumption (kWh), *E*_hour_ the average hourly electricity consumption (kWh h^−1^) calculated by dividing the measured energy use (kWh) by the monitoring duration (h), and *t*_annual_ the annual operating time (h). The measured equipment categories are listed below. The assessment excluded the cold room (2–8 °C) and research-specific instruments other than high-performance liquid chromatography (HPLC) systems, so the energy analysis is only partially complete. Nevertheless, we focused on equipment that is both highly energy-intensive and most numerous.

• Stoves: for high-temperature units (180 °C), two 24 hour measurements were performed to capture warm-up and steady-state consumptions, revealing only a 3% difference. Consequently, all remaining units were assessed with a single 24 hour measurement. For baseline, stoves were operated continuously (24 h × 365 days), while in the post-intervention phase, when stoves were switched off by default, annual operating time was estimated from documented usage schedules.

• Cold storage equipment: representative units varying in age (purchased between 2004 and 2022), size, and door-opening frequency were assessed. The sample included refrigerators (*N* = 6), freezers (*N* = 6), refrigerator–freezer combinations (*N* = 3), and ultra-low temperature (ULT) freezers (*N* = 2). Measurements were conducted in lab rooms with controlled ambient temperature (∼22 °C) and recorded over periods ranging from 48 h to 264 h per unit. Cold storage units were operated continuously (24 h × 365 days). For each equipment category, the mean energy consumption was used to estimate annual energy use, which was then extrapolated to the total number of units. Average annual energy consumption of refrigerators = 166 kWh; freezers = 458 kWh; combined refrigerator-freezer units = 240 kWh; ULT-freezers = 5495 kWh.

• Biosafety, polymerase chain reaction (PCR) and laminar airflow (LAF) cabinets: annual operating times were estimated from equipment booking logs using a stratified sampling approach. Representative weeks from the regular academic period (42 weeks) and summer period (8 weeks) were selected, with weekly averages calculated over four weeks and extrapolated to their respective durations to yield a weighted annual value.

• CO_2_ incubators (33–37 °C, 5% CO_2_): representative units from two age cohorts were measured (2003–2015 and 2016–2025 models). The incubators operated continuously (24 h × 365 days).

• HPLC systems: the measurements were performed under idle and active conditions, the latter including operation with either the Evaporative Light Scattering Detector (ELSD) or Photodiode Array (PDA) detector. Operational times were estimated from booking data, extrapolated to annual values under the assumption of consistent use.

#### 2.1.5 Energy consumption of fume hoods

Energy consumption of variable air flow (VAV) fume hoods was quantified through the LEAF calculators. Input variables included sash height and width, which were measured using a measuring tape. Additionally, average face velocity was derived by calculating the mean of the recorded value during official inspection for each unit. For modelling the operational usage, it was assumed that sashes were open on average 2 h per day.^31^ The LEAF calculators serve as estimation tools to demonstrate impact trends rather than to produce accurate absolute measurements; in this study, they were used to estimate the energy consumption of fume hoods, which could not be measured directly using plug-in energy meters, relative to other equipment categories.

#### 2.1.6 Framework for estimating costs and emissions from energy consumption

Across all equipment categories, operational electricity costs and CO_2_e emissions were calculated by multiplying measured energy consumption with institution-specific parameters: an electricity tariff of 0.10 EUR per kWh and an emission factor of 0.19 kg CO_2_e per kWh. Where the LEAF calculator did not report direct energy use, values were back-calculated from total costs dividing by the tariff using eqn (2):

2 *E*_annual_ = Cost_annual_/Tariff

where *E*_annual_ is the annual electricity consumption (kWh), Cost_annual_ the annual costs provided by the LEAF calculators (EUR), and Tariff the electricity tariff (EUR per kWh).

#### 2.1.7 Classification of waste streams

The Px labs handle experimental work with biological and chemical hazards. Biological work is conducted under biosafety levels ML-I (minimal risk) and ML-II (moderate risk). For this study, waste was classified into two groups: hazardous (contaminated) and recyclable (non-contaminated). Hazardous waste was divided into solid and liquid fractions, with subcategories based on chemical or biological content and sharp items. Recyclable waste was split into plastic packaging, mixed rigid plastics, paper and carton and expanded polystyrene. Residual waste, glass, and cardboard from deliveries were excluded due to difficulties in quantification.

#### 2.1.8 Quantification of hazardous waste streams

Hazardous waste streams were quantified during both baseline and post-implementation phases to enable comparative analysis. For solid hazardous waste, bins were inspected weekly and recorded only when full, sealed, and ready for disposal; weights were measured using a standard scale. The tare mass of empty bins was not subtracted, as bins are incinerated with their contents by the external processor. Liquid hazardous waste was quantified by counting fully filled 10 L jerrycans, while sharps waste was tracked by recording the number of completely filled and sealed containers.

#### 2.1.9 Quantification of non-hazardous waste streams

Recyclable (non-hazardous) waste streams were quantified only during the post-implementation phase, as systematic recycling procedures were absent at baseline. Each stream was collected in designated bins and weighed weekly using a standard scale. To ensure accuracy, bins were tared prior to use, and recorded weights were corrected for tare mass.

#### 2.1.10 Qualitative data collection and assessment of waste separation practices

During both baseline and post-implementation phases, qualitative data on waste separation practices were collected through systematic photographic documentation of waste bin contents across all lab areas. The photographs were analyzed to identify recurring patterns of improper disposal. Misplaced items were classified into defined categories and manually counted. To ensure comparability, raw counts were normalized for differences in data collection frequency and lab occupancy. Image collection was conducted weekly for 10 weeks during the post-implementation phase and bi-weekly for 8 weeks during the baseline phase; baseline counts were therefore multiplied by 2.5 to ensure comparability. Post-implementation frequencies were additionally normalized and extrapolated to the baseline number of lab users.

#### 2.1.11 Framework for estimating costs and emissions from waste production and processing

The financial assessment of hazardous waste disposal was based on pricing data from Utrecht university's waste processor (Table S3). For solid hazardous waste, costs were calculated by multiplying the category-specific price per kilogram by the total mass. For liquid hazardous waste, representative average container weights were determined by sampling multiple containers per category, and these averages were used to calculate disposal costs. The carbon footprint of hazardous waste processing was estimated following the methodology of Petry, 2025,^32^ which applied a screening life-cycle assessment (LCA) based on IPCC Guidelines for National Greenhouse Gas Inventories.^33^ Each waste category was assigned a carbon content fraction derived from default degradable organic carbon (DOC) and fossil carbon values specified by the IPCC guidelines (Table S2).^32^ CO_2_ emissions were estimated under the assumption of complete oxidation upon incineration. The calculation followed the carbon mass balance approach using the following equations:*C*_Mass_ = *Q*_Waste_ × *C*_Content_CO_2_ = *C*_Mass_ × 3.67where *C*_Mass_ equal the total carbon mass in waste (kg), *Q*_Waste_ equal the total quantity of waste (kg), *C*_Content_ is the assigned carbon fraction and 3.67 the molecular conversion factor from C to CO_2_ based on molar mass ratio (44 g mol^−1^: 12 g mol^−1^).^32^ Carbon emissions from hazardous waste transportation were excluded from this analysis due to the lack of disaggregated data by waste category or institutional site, making their integration into the carbon footprint model infeasible.

#### 2.1.12 Surveys

Adapting the survey from Schell, 2024,^23^ two questionnaires were designed using Qualtrics: a general survey for all division members and a targeted survey for LEAF-team members. Surveys were distributed *via* institutional email after the divisional LEAF-bronze accreditation: in February 2025 (Px division) and June–September 2025 (PCol division). The general survey was sent to all current members (Px: 55 employees, 27 interns; PCol: 73 employees, 30 interns). In total, 99 responses were received with overall response rate of 51% (57 Px, 70% response rate; 42 PCol, 41%). Only respondents with over six months of group membership could answer questions on perceived general changes. Questions on perceived laboratory changes were limited to those with over six months of membership who also conducted lab work at least once per month. The LEAF-team survey was sent to Px (13 members) and PCol (11 members), yielding a total of 18 responses with overall response rate of 75% (11 Px, 85%; 7 PCol, 64%).

## 3 Case study: lab sustainability at Utrecht university, results and discussion

### 3.1 Environmental footprint of wet labs at Utrecht university

**Fig. 1:**
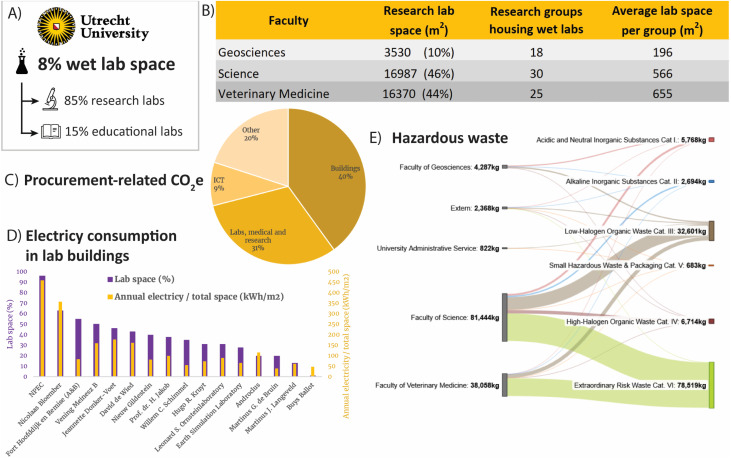
Overview of wet labs at Utrecht university and their partial environmental footprint. At Utrecht university, four of seven Faculties have lab facilities: geosciences, science, veterinary medicine, and medical sciences.^34^ Because the latter is managed by another organization (the University Medical Centre Utrecht), it was excluded from this analysis. (A) Percentage of university space occupied by wet labs, and the share of research and educational labs across the university. (B) Faculty-specific breakdown of research labs. (C) University-wide procurement-related CO_2_e emissions by sector (2023). (D) Annual electricity use (2024) per lab building normalized to total building area (yellow), ordered by decreasing lab space percentages (purple). (E) Annual amount of hazardous lab waste by faculty and waste category (2023).^32^

At Utrecht University, 73 research groups or divisions (*i.e.*, clusters of research groups functioning as a unit) operate lab facilities, averaging 505 m^2^ each. Of these, the Faculty of Science hosts the largest number of these research groups/divisions,^31^ followed by the Faculties of Veterinary Medicine^26^ and Geosciences.^18^ The Utrecht university-managed labs represent about 8% (43 541 m^2^) of the university's indoor space, of which 15% is dedicated exclusively to education, and 85% is primarily used for research by staff and students (Fig. 1A). When considering this second category (research labs), the Faculties of Science and Veterinary Medicine account for similar shares (46% and 44%, respectively), while geosciences houses a smaller share (10%) (Fig. 1B). Collectively, the relatively small fraction of Utrecht university's indoor space that is used as lab space accounts for a large fraction of organizational footprint due to the factors outlined below.

#### 3.1.1 Purchase of lab equipment and supplies

In accordance with the greenhouse gas (GHG) protocol,^35^ Utrecht university has committed to assessing its carbon footprint across all three scopes: Scope 1 (direct emissions), Scope 2 (indirect emissions from purchased energy), and Scope 3 (other indirect emissions, including procurement, travel, commuting, construction, waste, and property management). Carbon dioxide equivalent (CO_2_e) emissions express the combined global warming impact of all GHGs in terms of the amount of CO_2_ that would produce the same effect. In line with other scientific organizations,^8^ procurement is one of the largest contributors to CO_2_e, with purchases of research and lab items accounting for 31% of CO_2_e emissions (Fig. 1C). Moreover, Utrecht was the first Dutch university to conduct a biodiversity footprint analysis,^36^ building on earlier studies by Oxford University.^14^ Although methods are still evolving, procurement, particularly of lab supplies, emerges as one of the dominant drivers of biodiversity loss, exceeding impacts from international travel, electricity, or construction materials. For example, personal protective equipment relies on hydrocarbon extraction and processing, often sourced from biodiversity-rich regions.^14^ Similarly, Utrecht University's expenditure-based analysis identifies the purchase of lab items as the most significant contributor in the procurement sector, led by chemicals (notably helium, due to high volumes), followed by equipment (especially specialized devices with embedded electronics), and consumables.^36^

#### 3.1.2 Lab equipment and energy consumption

Labs are significant drivers of institutional energy demand, typically consuming far more than office spaces due to energy-intensive systems such as ventilation, cold storage, and specialized equipment.^10^ In 2024, Utrecht university operated 705 fume hoods and 530 fire safety cabinets, both requiring continuous airflow. Ventilation systems account for 25–70% of electricity consumption in lab buildings.^23^ A single fume hood can use up to 35 MWh per year,^37^ equivalent to the annual electricity consumption of approximately 12 Dutch households.^38^ Energy demand varies substantially by system type: constant air volume (CAV) systems maintain a fixed airflow, while newer variable air volume (VAV) systems automatically adjust ventilation rates based on use, resulting in significant energy savings. Cold storage was also substantial: the Faculty of Veterinary Medicine alone operated 82 ultra-low temperature (ULT) freezers, 199 standard freezers, and 286 refrigerators. A ULT freezer set to −80 °C typically uses ∼6 MWh year^−1^ [3–13.6 MWh year^−1^],^39,40^ equivalent to ∼2 Dutch households.^38^ Assessing total lab energy use is difficult, as reporting is generally at the building level. To understand lab contributions, we analyzed building-level electricity consumption relative to the proportion of lab space, normalized by floor area. As shown in Fig. 1D, buildings with a higher share of labs display higher normalized electricity use. In addition, detailed analysis of pharmaceutics (Px) division labs (see Section 2.2.1) revealed annual consumption of ∼400 kWh m^2^, well above both the research building average where they are located (∼170 kWh m^2^) and the Utrecht University average (∼70 kWh m^2^). These results align with previous studies highlighting the disproportionate energy intensity of lab environments.^10^

#### 3.1.3 Hazardous waste

The predominant hazard type in labs depends on the research focus. For example, the faculty of veterinary medicine produces primarily biological hazardous waste, geosciences mainly chemical, and science a mix of both. Hazardous waste includes both inherently hazardous materials and those contaminated with hazardous substances, with nearly all categories originating from wet labs and veterinary hospital (Fig. 1E and Table S1). In 2023, the Faculty of Science generated the most hazardous waste (64.1%), followed by veterinary medicine (30.0%) and geosciences (3.4%). Category VI (biologically contaminated waste, *e.g.*, genetically modified organism (GMO) and veterinary hospital waste) dominated with 78.5 tons (61.8%, with processing costs of ∼43 000 EUR), arising only from Science and Veterinary Medicine. Category III (low-halogen organic liquids and chemically contaminated solids) was next at 32.6 tons (25.7%, ∼30 000 EUR processing costs), with other solvents and solution wastes comprising 12.5%. Hazardous waste has a disproportionate environmental impact due to its reliance on high-temperature incineration, which drives substantial GHG emissions. Solid hazardous waste, largely composed of high-carbon polymeric materials, degrades into CO_2_ during combustion or gasification, contributing 28–54% of the life-cycle emissions of common lab consumables.^41–43^ Although hazardous waste represents ∼10% of Utrecht University's total waste volume, it accounts for ∼40% of waste-related CO_2_ emissions (∼175 t CO_2_ in 2023), since over 90% is currently incinerated.^32^

Overall, the environmental impact of Utrecht university labs is similar to that observed at other universities,^5–7,17^ underscoring the need for sustainability measures to reduce resource use and waste.

### 3.2 LEAF-bronze implementation in the department of pharmaceutical sciences

**Fig. 2:**
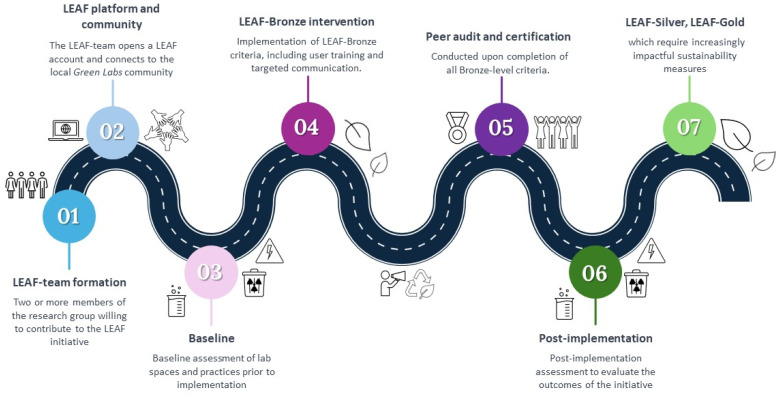
Roadmap for lab sustainability initiatives at Utrecht university using LEAF as the primary implementation framework. (1) Team formation: a LEAF-team is initiated by Green Team members and joined by staff or students committed to improving lab sustainability. (2) Registration: the LEAF-team registers the lab facility on the LEAF platform to access resources and connects with institutional or national Green Labs networks. (3) Baseline assessment: the team evaluates current lab practices, including space use, occupancy, waste streams, and equipment-related energy consumption. (4) Intervention: sustainable practices are introduced to meet LEAF-bronze criteria, supported by training and communication to engage all lab users. (5) Peer audit and certification: peer review confirms completion of all criteria, granting the LEAF-bronze award, which must be renewed annually. (6) Post-implementation assessment: the team measures environmental and social impacts. (7) Progression: divisions may then advance to Silver and Gold levels, requiring increasingly ambitious actions. Although baseline and post-implementation assessments were not mandatory to reach certification, they were conducted here to evaluate intervention outcomes.

Sustainability efforts in the department of pharmaceutical sciences (Faculty of Science) started in 2021 with the formation of a Green Team, initially focusing on educational curricula and teaching labs. More recently (early 2024), research staff started implementing the LEAF framework across all four lab divisions, of which two achieved LEAF-bronze (entry-level) certification, pharmaceutics (Px) and pharmacology (Pcol) in 2024 and 2025, respectively. LEAF-bronze emphasizes foundational measures, including switching off idle equipment, optimizing cold storage use, improving waste segregation and recycling and raising user awareness through signage and training.^18^ It also requires documenting practices and assigning responsibility, forming a basis for more advanced interventions. This section reports the main findings on environmental (Px only) and social outcomes (Px and PCol) of LEAF-bronze intervention, which followed the steps illustrated in Fig. 2.

#### 3.2.1 Equipment and energy assessment

**Fig. 3:**
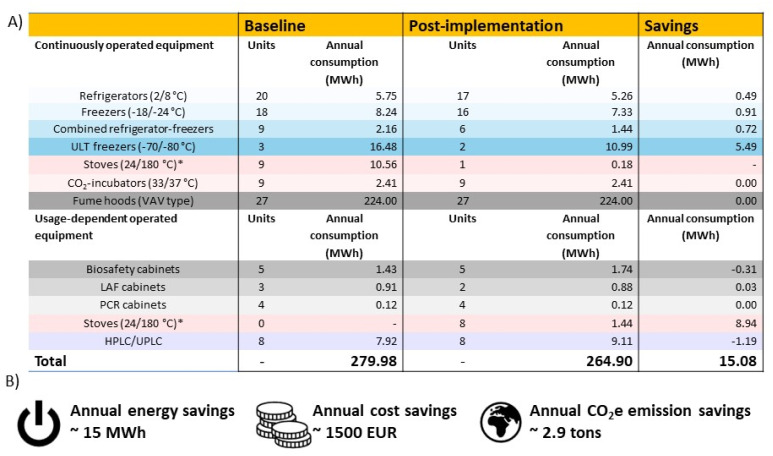
Electricity consumption and savings following LEAF-bronze interventions in Px labs. (A) Equipment categories, unit counts, and annual energy consumption before and after implementation, highlighting electricity savings. Equipment is grouped into continuously operated and usage-dependent categories. Color code: blue = cold storage equipment (refrigerators, freezers, combined refrigerator–freezer units, and ULT freezers); red = heat-generating equipment (stoves and CO_2_ incubators); gray = ventilation systems (fume hoods, biosafety cabinets, polymerase chain reaction (PCR) cabinets, and laminar airflow (LAF) hoods); purple = research-specific equipment (high-performance liquid chromatography (HPLC) systems). *All stoves were continuously turned on at baseline but most were switched to OFF-by-default with a usage log after the intervention. (B) Annual electricity savings and corresponding reductions in operational costs and CO_2_e emissions.

The Px lab facility contains a wide range of equipment, with the main ones reported in Fig. 3A. Targeted LEAF-bronze measures improved equipment efficiency and reduced energy use (Fig. 3). A comprehensive cleanup and space reorganization of cold storage enabled the decommissioning of nine old, thus inefficient units (three refrigerators, three combined units, two freezers, and one ULT freezer) and storage temperatures were raised within allowable ranges. In addition, stove operation was minimized by switching eight out of nine units, previously left on continuously, to an OFF-by-default mode supported by a usage log (Fig. 3A). Surprisingly, a stove operating at 180 °C (∼3.5 MWh year^−1^) consumed a similar amount of electricity as a ULT freezer at −80 °C (∼5.5 MWh year^−1^). These interventions reduced total Px lab electricity use by ∼5%, while for cold storage and ovens alone the energy consumption declined by 38%, leading to proportional decreases in CO_2_e emissions and costs (Fig. S1). In our case, the largest savings came from stoves (8.94 MWh year^−1^), followed by ULT freezers (5.49 MWh year^−1^) and other cold storage units. Overall, the measures reduced emissions by ∼2.9 t CO_2_e, saving roughly 1500 EUR per year on direct energy costs alone. Additional actions, such as equipment labeling and optimized monitor settings, further promoted awareness, although their impact was not quantified. Consistent with previous findings,^7,18^ the results demonstrate that LEAF-bronze measures can deliver substantial environmental benefits at minimal effort. Based on the savings achieved by Px division (representing ∼2% of Utrecht university's total lab space), scaling up the LEAF-bronze accreditation across all Utrecht university labs could yield cumulative annual energy savings of approximately 750 MWh, equivalent to 75 000 EUR. Moreover, ventilation systems, accounting for approximately 80% of energy use in Px labs, were not systematically addressed by LEAF-bronze intervention but will be covered in higher certification levels, highlighting their importance for maximizing future impact.

#### 3.2.2 Waste assessment

**Fig. 4:**
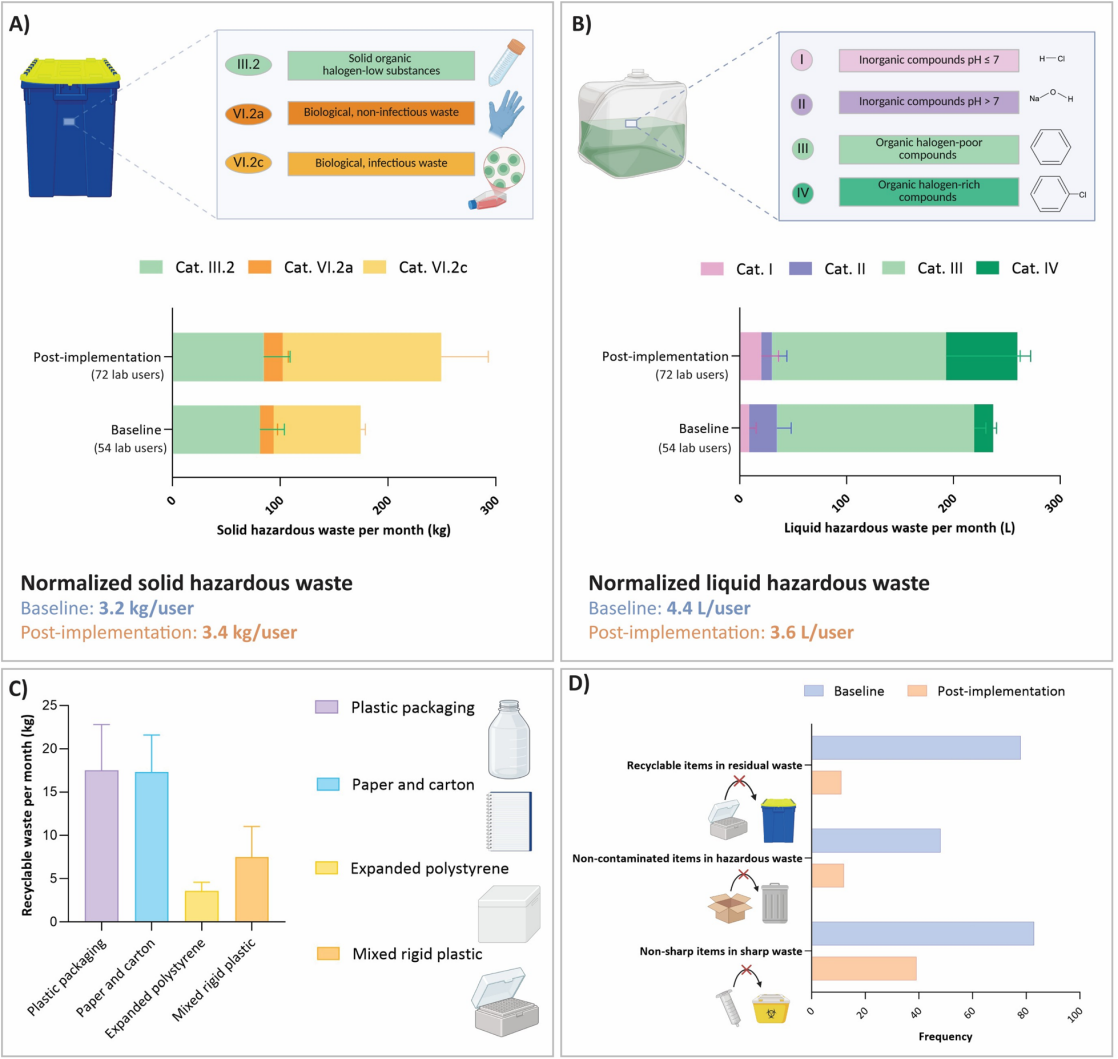
Waste generation and sorting practices in Px labs before and after LEAF-bronze implementation, with each recording phase spanning the same three months one year apart. (A) Average monthly solid hazardous waste. (B) Average monthly liquid hazardous waste. (C) Average monthly recyclable (non-contaminated) waste collected post-implementation. Glass and cardboard from delivery boxes were not quantified because reliable weighing was not feasible during the study period. (D) Frequency of improper waste sorting recorded through photographic documentation throughout each phase.

Before LEAF interventions, each lab room had bins for solid and liquid hazardous waste, sharps, and residual waste, while local recycling collection points were limited to a few hallway containers (plastic packaging, paper, glass) or absent (mixed rigid plastics and expanded polystyrene). Although users received verbal instructions for waste disposal practices, the complexity of waste streams led to confusion and thus frequent mis-sorting, such as non-sharps in sharps bins, recyclables in residual waste, and clean items in contaminated waste (Fig. 4D). The LEAF bronze-level strategy significantly improved sorting practices, reducing the frequency of mis-sorting (Fig. 4D). Interventions included clearly labeled, accessible bins, standardized signage, and marked collection points, with emphasis on recyclables but improved hazardous waste segregation as well. Guidance was reinforced through waste flow charts (Fig. S2), illustrated guidelines and training *via* divisional presentations and an interactive quiz, engaging both new and existing users. Importantly, all measures were implemented in collaboration with the central waste department to ensure accuracy and compliance with organizational guidelines.

During baseline and post-implementation phases (three months each), solid and liquid hazardous, as well as recyclable waste, were quantified (Fig. 4A–C). Sharps waste was excluded because the measurement period was too short to yield significant volumes. Results were normalized to user numbers, which rose by 33% after LEAF implementation (54 to 72; Fig. S3). Solid hazardous waste increased from 524 to 749 kg, raising the monthly division-wide average from 175 to 250 kg. Normalized per user, however, generation remained stable at ∼10 kg per three months per user (∼3 kg per month per user). ML-II labs were the largest contributors, producing about twice the divisional per-capita average (Fig. S4A and B), reflecting reliance on single-use plastics.^13^ Notably, full ML-level bins weighed less than those from non-ML labs (5.5 *vs.* 7.8 kg on average), suggesting the presence of more bulky, low-density items such as cell culture flasks. Liquid hazardous waste rose from 712 to 780 L, increasing the monthly average from 237 to 260 L. Per user, volumes decreased slightly (13 L to 11 L; ∼4 L month^−1^). Although liquid hazardous waste composition varied by lab area between phases, driven by differences in experimental activities, category III (halogen-poor organic solvents) dominated both phases, with the chemistry-focused lab as the main contributor (Fig. S4C–F). Post-implementation analysis showed that 78% of its liquid waste came from solvent-intensive purification methods *via* dialysis (Fig. S4G). User training during the intervention, which emphasized minimizing solvent volumes, likely contributed to the reduction in liquid waste in the chemistry lab observed post-implementation (∼35% overall; ∼50% normalized per user). Waste-related CO_2_ emissions were highest for category III liquid waste (62% baseline; 45% post), but costs were dominated by solid chemical waste due to its high processing fee (III.2: 67%/63%; Tables S2 and S3). Recyclable waste was tracked only post-implementation, as no systematic recycling existed at baseline (Fig. 4C). Plastic packaging (∼18 kg month^−1^) and paper/carton (∼17 kg month^−1^) waste were most abundant, followed by rigid plastics (∼8 kg month^−1^) and expanded polystyrene waste (∼4 kg month^−1^). Use of plastic packaging was highest in ML-I/II labs, reflecting the consumption of single-use plastics, while paper/carton waste levels were consistent across lab areas (Fig. S4H and I).

In conclusion, hazardous waste volumes remained largely unchanged as LEAF-Bronze interventions focused on improving sorting and expanding recycling rather than reducing waste generation. However, chemistry-focused lab training on solvent use likely contributed to reducing liquid waste in that area, which dropped by 50% per user. The greatest mitigation potential lies in solid waste from biology labs (ML-I/II) and organic liquid waste from chemistry labs, addressed in the further LEAF-levels. Sorting practices improved, and substantial recyclables were collected post-implementation, but ongoing training and reinforcement are needed since mis-sorting, though reduced, persists (Fig. 4D).

#### 3.2.3 General satisfaction surveys

**Fig. 5:**
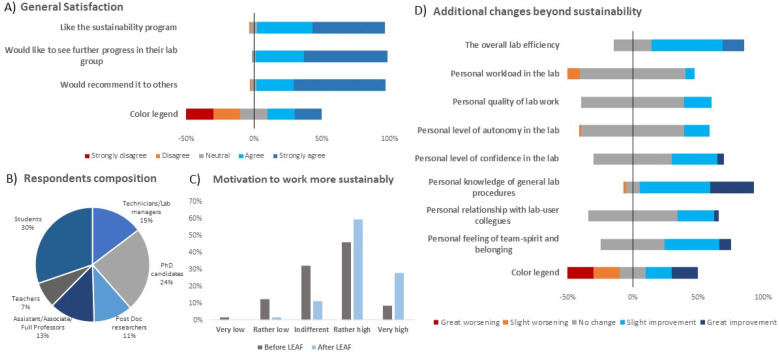
Survey results on satisfaction with the lab sustainability initiative following LEAF-bronze implementation in the Px and Pcol research divisions. (A) Responses to general satisfaction questions about the sustainability program (*N* = 99). (B) Demographic composition of respondents (*N* = 99; 57 Px, 42 Pcol, 20% LEAF-team members). (C) Motivation to adopt sustainable practices in the lab before and after LEAF-bronze implementation. Inclusion criterion: lab membership > 6 months (*N* = 72; 34 Px, 38 Pcol). (D) Perceived changes beyond sustainability. Inclusion criterion: lab membership > 6 months and regular lab work (*N* = 57; 28 Px, 29 Pcol).

Following LEAF-bronze certification, all Px and Pcol division members were surveyed to assess satisfaction, motivation for sustainable practices, and perceived benefits and impacts (Fig. 5). A total of 99 responses were collected (51% response rate), with respondent composition shown in Fig. 5B and including 20% active LEAF-team members and 5% former members who left mainly due to time constraints. Overall, the initiative generated high satisfaction across both Px and Pcol divisions. Most participants viewed the program positively (40% “somewhat”, 54% “a great deal”) and supported its continuation (35% “somewhat”, 62% “a great deal”), with 95% recommending adoption elsewhere (27% “probably”, 68% “definitely”) (Fig. 5A). Respondents with more than six months in their division, who had experienced both pre- and post-LEAF conditions, reported increased motivation to adopt sustainable practices (Fig. 5C). Beyond environmental benefits, participants cited added value, such as improved lab organization (83%), process optimization (51%), stronger team cohesion (44%), and enhanced safety (40%). Comments also mentioned financial savings and better alignment with grant requirements. Regarding impacts in lab work, respondents reported gains in lab efficiency, knowledge of general practices, and sense of belonging. More modest improvements were seen in confidence and relationships with colleagues, while workload, work quality, and autonomy showed little change (Fig. 5D). Consistent with previous findings,^23^ this survey indicated high overall satisfaction, increased motivation, and perceived benefits extending beyond sustainability.

#### 3.2.4 LEAF-team members' surveys

**Fig. 6:**
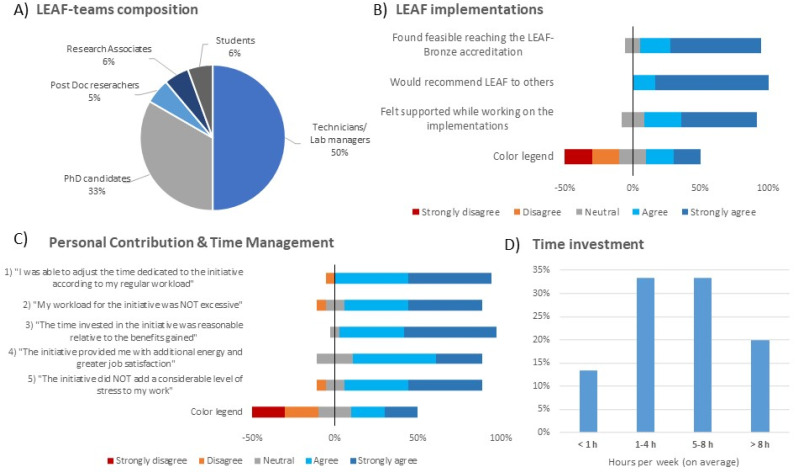
Survey results on LEAF-team members' experiences with the lab sustainability initiative in the Px and Pcol divisions. (A) Demographic composition of respondents (*N* = 18; 11 Px, 7 Pcol). (B) General experiences with LEAF-bronze implementation. (C) Perceptions of individual contribution and time management. (D) Estimated time investment over the ∼10 month implementation period.

Following LEAF-bronze certification, a targeted survey of LEAF-team members in the Px and Pcol divisions was conducted to assess motivations, time commitment, experiences, and feedback on LEAF as a framework (Fig. 6). Among the 18 respondents (75% response rate), most were technicians/lab managers (50%) or PhD candidates (33%) (Fig. 6A). Two-thirds had been involved for over 12 months, and all joined voluntarily, as sustainability was not formally part of their job. Some technicians noted indirect alignment with their duties through improved lab efficiency and organization. The main motivations for joining were reducing labs' environmental footprint (56%) and improving efficiency (39%), with additional drivers including personal interest in sustainability (22%), urgency for climate action (22%), recognition of current wasteful practices (17%), and curiosity about improvements (11%). Motivation remained strong for all, with 22% reporting increased levels after certification. Most intended to continue (67% “definitely”, 17% “probably”), with departures linked only to contract completion. Most respondents considered achieving LEAF-bronze feasible (22% “quite”, 67% “very”) and felt well-supported (28% “quite”, 56% “very”) through the LEAF platform, the Green Team, the national Green Labs network, and peer guidance (Fig. 6B). While divisional management support was acknowledged, stronger faculty- and university-level backing, such as a central LEAF coordinator, was recommended. Expanding the LEAF team was also suggested to allow a more equitable distribution of tasks. All respondents would recommend LEAF (17% “probably”, 83% “definitely”), but emphasized that certification should not be the ultimate aim, as the primary goal is reducing environmental impact. They noted that LEAF (bronze) provides structure but does not cover all interventions, particularly those requiring institutional action or more support for efficient data management. Participation enhanced job satisfaction and energy for many (50% moderate, 28% substantial), without excessive workload or stress (44% “not at all”, 39% “a little”) (Fig. 6C). Regarding workload, most could adjust their time commitment with ease (44% “quite”, 50% “very”) and found the time investment reasonable relative to the achieved benefits (39% “quite”, 56% “very”). Regarding time investment (Fig. 6D), 56% of respondents reported difficulty with accurately estimating their average monthly contribution, as 11% did not provide a value. Another 11% noted that their time commitment varied considerably over the initiative period. Over a ∼10 month period (baseline plus implementation), weekly time investment was <1 h (13%), 1–4 h (33%), 5–8 h (33%), or >8 h (20%).

### 3.3 Towards LEAF-bronze for all labs and beyond: future directions for lab sustainability at Utrecht university

Driven by growing evidence, university-wide Green Labs Task Force advocacy, and internal case studies (presented in Section 2.2), Utrecht University achieved significant progress in 2025: all faculties with labs and Facility Management formally committed to lab sustainability, appointed dedicated coordinators, and integrated the goal of achieving LEAF-bronze (entry level) certification for all labs by 2030 into the Strategic Plan, marking a key milestone in institutionalizing sustainable lab practices.

This section outlines Utrecht university's ongoing efforts to extend sustainable lab practices across the institution, emphasizing the integration of bottom–up initiatives with top–down support, as well as current challenges and future directions.

#### 3.3.1 Community support and engagement

In line with previous studies,^7,16^ successful implementation of both user-level and systemic sustainability measures depends on strong community support, clear communication, shared goals, and collective action. Recently, at Utrecht university, we foster these aspects through (i) regular meetings and communication platforms for engaged lab users (*e.g.*, LEAF teams); (ii) institutional and departmental sustainability coordinators to provide guidance and track progress; and (iii) interdisciplinary committees, such as the university-wide Green Labs Task Force, to connect stakeholders. The latter two are especially important for initiatives requiring central coordination and consistent execution, such as standardized recycling and energy-efficient ventilation systems. Moreover, including lab personnel in decision-making committees as lab stewards ensures that practical expertise informs policy.

Engaging all lab personnel is crucial for the successful implementation and long-term maintenance of interventions, supported by continuous user training to address high staff turnover. Although creative strategies like Freezer or Data Clean-up Challenges can motivate participation and yield significant benefits,^12^ the institutional integration of sustainability frameworks, such as LEAF, or the development of tailored ones, remain essential for broad participation and long-term continuity. Moreover, those committing efforts (*e.g.*, LEAF teams) should be recognized or rewarded, and time should be granted to prioritize sustainability activities. For example, PhD candidates from Utrecht University's Graduate School of Life Sciences participating in lab sustainability activities can now obtain credits for their mandatory training curriculum.

Behavioral change and cultural shifts at the user level remain major challenges. Our experience in the pharmaceutical sciences department indicated that small, incremental steps, continuous communication, and strong team spirit are key to success. According to responses in our surveys, the most difficult aspect was ensuring that all lab members adapted their habits to align with the new system, which required sustained motivation and support. Management support proved essential in communicating the reasons for changes, ensuring adherence, and fostering accountability. Success depended on involving everyone, yet task organization and coordination remained challenging given the number of people required. Within grassroots LEAF teams, efficient structuring of responsibilities could further enhance implementation.

#### 3.3.2 Energy efficiency and reduction

Consistent with previous studies,^7,12,18^ our findings showed that substantial energy savings can be achieved through researcher-driven initiatives without major infrastructural changes. Ventilation systems and cold storage are among the most abundant and energy-intensive equipment and key targets for sustainability efforts. Cold storage efficiency can be improved through regular defrosting, removal of outdated samples, proper inventory management, and temperature optimization. Freezer challenges set up by various organizations (*e.g.*, international: My Green Lab and national: Green Labs NL), highlight the impact of such actions.^44^ Adjusting ULT freezers from −80 °C to −70 °C can reduce energy use by about 30% without affecting sample integrity, as confirmed by multiple stability studies.^7,45–47^

Significant efficiency gains can also be achieved in ventilation systems through improved user practices and technical interventions. At the researcher level, programs like Harvard University's Shut the Sash encourage closing fume hood sashes when not in use, saving about US$200 000 annually and cutting CO_2_e emissions by over 300 tons.^31^ Avoiding long-term storage inside fume hoods is equally important to maintain proper airflow and reduce energy demand. The LEAF framework incorporates these practices: bronze level covers user behavior, while silver and gold levels address air change rate optimization and fume hood performance. Facility-level interventions further enhance efficiency, such as modifying ductwork (*e.g.*, reducing diameter or optimizing plenum design) can lower static pressure and energy demand.^48^ Likewise, adjusting conservatively high air change rates can yield large savings without compromising safety; reducing rates from 23 to 12 air changes per hour can lower energy use by 35% while maintaining stable negative pressure.^49^

A key limitation of researcher-driven initiatives is that financial savings often accrue centrally rather than to the research groups implementing them. Introducing reward mechanisms, such as reinvesting part of the savings to replace inefficient equipment, could strengthen motivation and sustain engagement.^50^ Moreover, existing guidelines and legislation can restrict the adoption of energy-efficient practices; for example, clinical study samples in ULT freezers must be stored at −80 °C, preventing adjustments to higher temperatures.

#### 3.3.3 Sustainable data management

Beyond equipment and ventilation, certain labs, especially those in computational research, bioinformatics, and other data-intensive fields, consume substantial energy due to high-performance computing and data management demands.^51–53^ Dedicated servers, powerful graphic processing units (GPU), and continuous data storage significantly increase electricity use, while the cooling systems required to maintain optimal hardware temperatures add further load. As data generation and analysis grow in scale and complexity, the energy footprint of digital research operations is becoming a critical component of sustainable lab practices.^24,54^ To mitigate this impact, several guidelines and frameworks have been developed, such as the open-access Green DiSC certification,^25^ which offers tailored criteria for both research groups and central support teams (*e.g.*, sustainability team or IT departments). At Utrecht university, pilot initiatives have recently been launched to implement Green DiSC in both wet and computational labs.

#### 3.3.4 Resource circularity and waste reduction

Waste reduction is a key pillar of lab sustainability, though achieving savings is often more complex than reducing energy use due to the diversity of lab waste. Indeed, LEAF-teams at Utrecht university identified the creation of clear and comprehensive waste segregation guidelines as a major challenge. While disposal is strictly regulated, detailed knowledge of waste composition, especially for non-hazardous streams, combined with circular economy principles following the R-ladder hierarchy (rethink, reduce, reuse, repurpose, and recycle^55^), can significantly lower a lab's carbon footprint, as reflected in higher LEAF certification levels (silver and gold). Moreover, the principles of green chemistry further contribute by minimizing hazardous substance use.^56,57^ However, the wide variety of materials and hazard classifications limits the feasibility of one-size-fits-all solutions.

Consistent with our findings, the greatest recycling gains can be achieved with packaging waste, which can be further reduced through order consolidation and right-sized purchasing. Pilot recycling initiatives of plastic waste from biosafety level 1 labs have demonstrated feasibility,^7,13,20,58–60^ but broader adoption is constrained by regulatory complexity, differing recycling standards, and limited institutional support.^13^ Manufacturers' take-back schemes and start-ups developing low-emission plastic decontamination and recycling methods further highlight the potential in this area.^12^ Higher up the R-ladder, reuse and repurposing of consumables offer additional opportunities.^13^ Examples include plastic items, such as repurposing empty solvent jerrycans for liquid waste^7^ and reusing cell culture flasks. Documenting and sharing such non-standard practices can accelerate their wider adoption.^22^ At the “Reduce” level, substituting high-impact materials and optimizing experimental design and workflows can substantially limit waste generation.^7,13,19,58,61,62^ For instance, replacing conventional dialysis as a purification technique with tangential flow filtration (TFF) has the potential to cut solvent use significantly.^63^ However, implementing new, optimized procedures or products requires dedicated piloting, training, and time investment. Together, these approaches provide a comprehensive framework for minimizing the environmental footprint of hazardous and non-hazardous waste in lab environments.

#### 3.3.5 Sustainable procurement

Although the lab materials supply chain accounts for a major share of universities' procurement-related carbon and biodiversity footprints,^8,14,36^ implementing sustainable purchasing practices remains challenging. The complexity and limited transparency of product data and life cycle assessments (LCAs), which evaluate environmental impacts from raw material extraction to disposal, hinder informed decision-making. Procurement-related emissions vary widely across labs and disciplines and correlate strongly with budget size.^8^

Lab users often lack sustainability knowledge and purchasing is typically driven by cost and research-quality priorities rather than environmental performance. Tools such as EnergyStar or My Green Lab's ACT ecolabel allow comparison of lab product sustainability profiles, but users must review them carefully, as the label ensures transparency of emissions data rather than a guaranteed level of sustainability.^21^ Embedding such data into institutional procurement systems in an accessible format could guide purchasing toward more sustainable options. Certain animal-derived products used in life sciences, including fetal calf serum (FCS) and basement membrane extract (BME), present ethical concerns along with the sustainability challenges. Databases such as those developed by the 3R Centre at Utrecht science park increasingly support informed selection of more ethical and sustainable products.^64^ In addition, tenders and centralized procurement offer opportunities to integrate sustainability criteria, but environmental factors remain secondary to cost, quality, and delivery speed. Resistance may also arise from researchers concerned about performance and validation of unfamiliar products. Appointing sustainability experts with lab experience to procurement teams could help balance environmental and operational priorities.

As for waste mitigation, applying circularity principles can further enhance sustainable procurement. Utrecht university's biodiversity footprint analysis identified purchasing second-hand equipment as an especially effective strategy.^36^ Initiatives such as the Laboratory Exchange Platform for Utrecht Science Park (LABEX-USP) can strengthen local circularity by providing a digital marketplace for sharing, donating or renting surplus equipment and consumables.^65^ For full impact, such platforms should be integrated into standard procurement workflows. Universities are also collaborating locally to share procurement data and develop joint mitigation strategies. In line with previous studies showing up to 20% total emissions reductions, and up to 40% for procurement-related emissions,^8^ systemic, coordinated interventions are essential to reduce the carbon and biodiversity footprint of research procurement.

#### 3.3.6 Circularity in lab buildings and interior design

Regarding construction and renovation, lab buildings typically embody about twice the carbon of standard office buildings, largely because of the reinforced concrete and steel needed to meet vibration, load-bearing, and safety standards.^66^ Applying circularity principles to lab construction, particularly for installations and interior fittings, can significantly reduce impacts. Adaptive reuse of existing structures minimizes the demand for new materials, though converting offices or other facilities into labs requires careful assessment of structural and technical needs. When additional space is necessary, efficiency can be improved through shared facilities, centralized storage, and core labs serving multiple research groups or departments.^67^ Shared research spaces also reduce energy use, waste, and costs; for example, a University of Colorado Boulder case study reported annual savings of approximately US$253 000 through lower plug loads, reduced supply use, and less maintenance compared to individual labs.^68^ Utrecht University has committed to develop circular buildings that minimize new raw material use and maximize recycled content.^69^ This approach extends to lab renovations, new constructions, and maintenance, emphasizing the reuse of surplus furniture and specialized equipment from decommissioned labs.

#### 3.3.7 Legislation and compliance

Sustainable lab practices contribute to corporate sustainability reporting and often align with national and international health, safety, and environmental (HSE) regulations, such as improved waste segregation and user training. Sustainable lab practices support corporate sustainability reporting^70^ and often align with national and international health, safety, and environmental (HSE) regulations, including improved waste segregation and user training. For example, clearly labelled lab waste bins can increase awareness of hazards and safety, which are sometimes overlooked in practice. Similarly, reinforcing training on a risk-based glove policy promotes glove use based on task-specific risk assessment rather than automatically worn in all situations. Under this approach, gloves are worn only when a relevant hazard is present, reducing unnecessary use, improving hygiene awareness, and minimizing waste.^71^ Some implementations, like reducing ventilation rates, require careful re-evaluation of existing safety measures. In such cases, close collaboration with organizational and international HSE departments is essential to ensure thorough risk assessment and regulatory compliance. HSE departments can further support sustainability by integrating relevant criteria into their routine lab audits. For example, at Utrecht University, Faculty HSE officers will conduct annual audits of selected LEAF criteria after initial certification to ensure ongoing compliance and reduce the workload of sustainability coordinators. Ultimately, environmental sustainability parallels workplace safety but operates on a longer time scale, addressing the broader question: What are the risks to humanity if we fail to adapt our practices?

## 4 Conclusion

This Utrecht University case study reports the achievements, challenges, and lessons learned from implementing lab sustainability initiatives to support the global shift toward greener science. Consistent with previous studies,^7,23^ our findings showed that entry-level frameworks like LEAF-Bronze yielded significant environmental benefits while enhancing users' motivation and satisfaction. Implementing these interventions across all Utrecht University labs could yield cumulative annual savings of approximately 75 000 EUR in direct electricity costs alone. While LEAF-bronze offers a low-threshold, effective starting point, further actions, such as those outlined in the -silver and -gold levels, are required to actually meet sustainability goals. Moreover, to enable broader adoption, fee-free programs, unlike the university-bound LEAF, are needed. Therefore, we embrace the future transition to open-access frameworks, such as the Sustainable Practices and Resource Knowledge Hub (SPARK-Hub) currently in its pilot phase and supported by international research funding organizations.^72^ Ultimately, any sustainable effort can contribute to reducing the environmental impact of scientific research.

Successful implementation of lab sustainability relies on strong community support, both internal and external (*e.g.*, local, national, and international Green Labs networks), as well as shared goals and collective action. Broad participation across labs is essential, as collective adoption of simple actions yields greater impact than isolated, high-effort initiatives. Institutional integration of lab sustainability practices is therefore key to consistent and lasting progress. Progress is most effective when bottom–up initiatives, such as LEAF-teams, are integrated with top–down support from facility managers, research leaders, and institutional leadership.^16^ This top-down engagement can be reinforced through funding policies that set minimum sustainability criteria, as recently promoted by European agencies pursuing a holistic strategy.^26^ While certification schemes like LEAF empower lab users to adopt sustainable practices locally, many high-impact measures, such as ventilation upgrades or building-level policies, require centralized oversight. Even LEAF-bronze actions benefit from institutional coordination to ensure consistency and efficiency, for example, through standardized recycling systems and comprehensive waste-sorting guidelines managed at the organizational level rather than by individual labs. By embedding sustainable practices into the existing organizational structures through both bottom–up and top–down approaches, we aim to make them standard for research groups and operational management.

To achieve wider and long-lasting adoption of sustainable practices, a strong focus on education is also required: integrating sustainability into teaching labs and curricula, training educators, and engaging students as active participants in the transition towards sustainable science. Ultimately, embracing the “less is more” philosophy, reflected in the higher levels of the R-ladder, “Refuse” and “Rethink”, calls for re-evaluating how science is practiced. This shift means prioritizing quality over quantity, advancing open access and study reproducibility, and focusing on research that delivers tangible social impact. Scientists are uniquely positioned to lead this cultural and systemic transformation; if the scientific community does not set the example, it is hard to imagine who else will.

## Author contributions

G. D. M. and E. J. H. conceptualized the research project and coordinated it under the supervision of T. V, R. M. and J. R. C. The research was conducted by G. D. M., E. J. H. and J. W. R. with the support of M. J. S., K. A. T. X., A. D. L, B. S. and J. S. The manuscript was prepared by G. D. M. with input from all other authors. All authors reviewed the manuscript.

## Conflicts of interest

There are no conflicts to declare.

## Supplementary Material

RA-016-D6RA04362C-s001

## Data Availability

The data that support the findings of this study are available from the corresponding author upon reasonable request. Ref. 73 are cited in the supplementary information (SI). Supplementary information is available. See DOI: https://doi.org/10.1039/d6ra04362c.
