# Supplementary material for: From grassroots to strategy: advancing laboratory sustainability at Utrecht university
Source: RSC Adv. 2026 Jun 2;16(32):29791–806. doi: 10.1039/d6ra04362c (PMC13231363; doi:10.1039/d6ra04362c)
Supplement: RA-016-D6RA04362C-s001 [file RA-016-D6RA04362C-s001.pdf]

Supplementary Information (SI) for RSC Advances.
This journal is © The Royal Society of Chemistry 2026

Supplementary Information

Supplementary Tables

Table S1. Hazardous and non-hazardous waste categories with corresponding Utrecht University classifications and EURAL codes from the European Waste Catalogue (EWC) [73]. For hazardous waste, category V (batteries, polishes) was excluded, as it does not originate from wet labs. It consists of nearly 0.5 % of total hazardous waste produced at Utrecht University (2023).

Hazardous waste	Category	EURAL code
Liquid acidic and neutral inorganic substances	Cat. I	20 01 14
Liquid alkaline inorganic substances	Cat. II	20 01 15
Liquid halogen-poor organic waste substances	Cat. III	20 01 13
Solid organic halogen-low substances	Cat. III.2	15 02 02
Liquid halogen-rich organic waste substances	Cat. IV	14 06 02
Biological, non-infectious substances (GMO)	Cat. VI.2a	18 01 03
Biological, infectious substances (GMO)	Cat. VI.2c	18 01 03
Sharp items	-	18 01 06
**Non-hazardous waste**		
Plastic packaging	-	15 01 02
Mixed rigid plastics	-	20 01 39
Paper and carton	-	20 01 01
Expanded polystyrene	-	17 02 03
Glass	-	20 01 02
Residual waste	-	20 03 01

Table S2. Monthly carbon emissions related to the processing of hazardous waste produced during baseline and post-implementation phases in Px labs. Categories I and II were excluded, as their aqueous/inorganic composition contains negligible carbon and they are processed by precipitation rather than incineration.

Hazardous waste category	Emission factor per category (kg CO_2 /kg waste)	Baseline CO_2 emissions (kg)	Post-implementation CO_2 emissions (kg)
**Cat. III**	2.596	475	420
**Cat. III.2**	1.835	149	155
**Cat. IV**	2.569	45	171
**Cat. VI**	1.101	103	181
**Total**		772	927

Table S3. Monthly costs per waste category for the processing of hazardous waste produced during baseline and post-implementation phases in Px labs.

Hazardous waste category	Cost per category (EUR / kg waste)	Baseline costs (EUR)	Post-implementation costs (EUR)
**Cat. I**	1.58 (2024); 1.67 (2025)	14	33
**Cat. II**	0.99 (2024); 1.05 (2025)	26	11
**Cat. III**	0.75 (2024); 0.79 (2025)	139	129
**Cat. III.2**	6.00 (2024); 6.02 (2025)	489	510
**Cat. IV**	0.45 (2024); 0.47 (2025)	8	31
**Cat. VI**	0.54 (2024); 0.57 (2025)	53	93
**Total**		**729**	**807**

Supplementary Figures

A)

	Baseline	Post-implementation
**Annual consumption (MWh)**	43.20	26.63
**CO_2e emission (t)**	8.21	5.06
**Electricity costs (EUR)**	4320	2663

B)

**Annual CO_2e emission (t)**	**Baseline**	**Post-implementation**
Refrigerators	1.09	0.99
Freezers	1.56	1.39
Combined refrigerator-freezers	0.41	0.27
ULT- freezers	3.13	2.08
Stoves	20.06	3.08

C)

**Annual electricity cost (EUR)**	**Baseline**	**Post-implementation**
Refrigerators	575	526
Freezers	824	733
Combined refrigerator-freezers	216	144
ULT- freezers	1648	1099
Stoves	3132	2088

Figure S1. Overview of **A)** annual electricity consumption, costs, and CO_2e emissions for cold storage equipment and stoves before and after implementation in the Px labs; **B)** annual CO_2e emissions by equipment category; and **C)** annual electricity costs by equipment category.

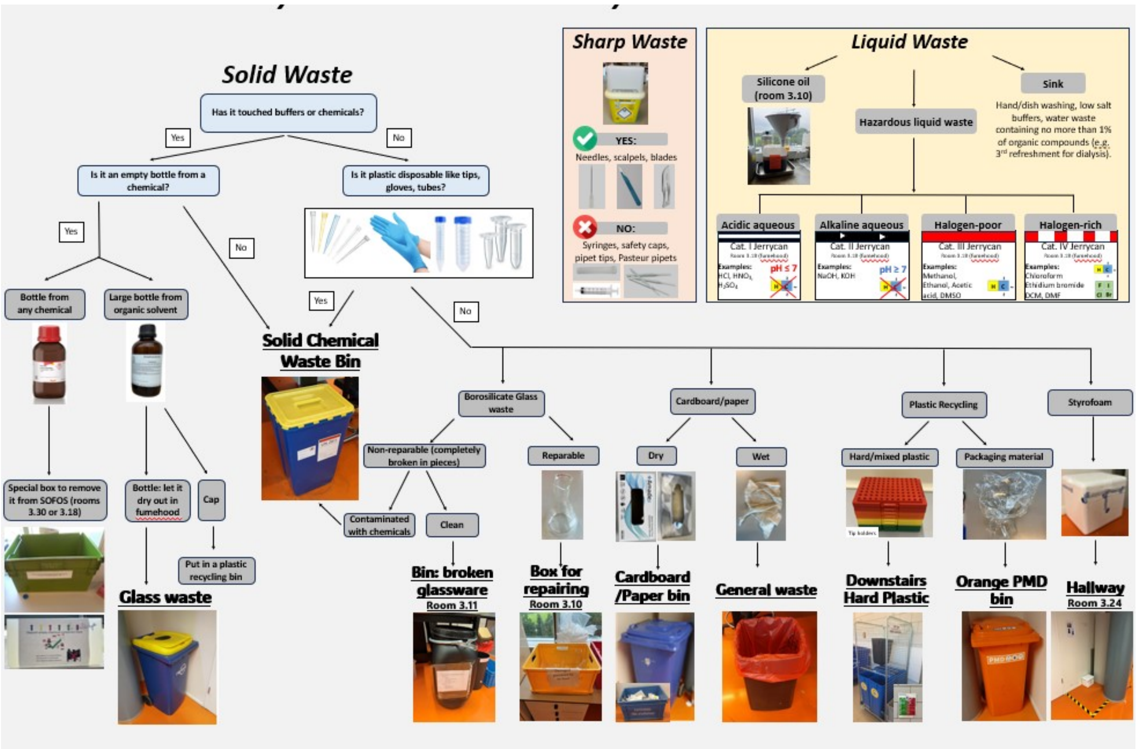

Figure S2. Example of decision flow chart for lab waste used in Px labs

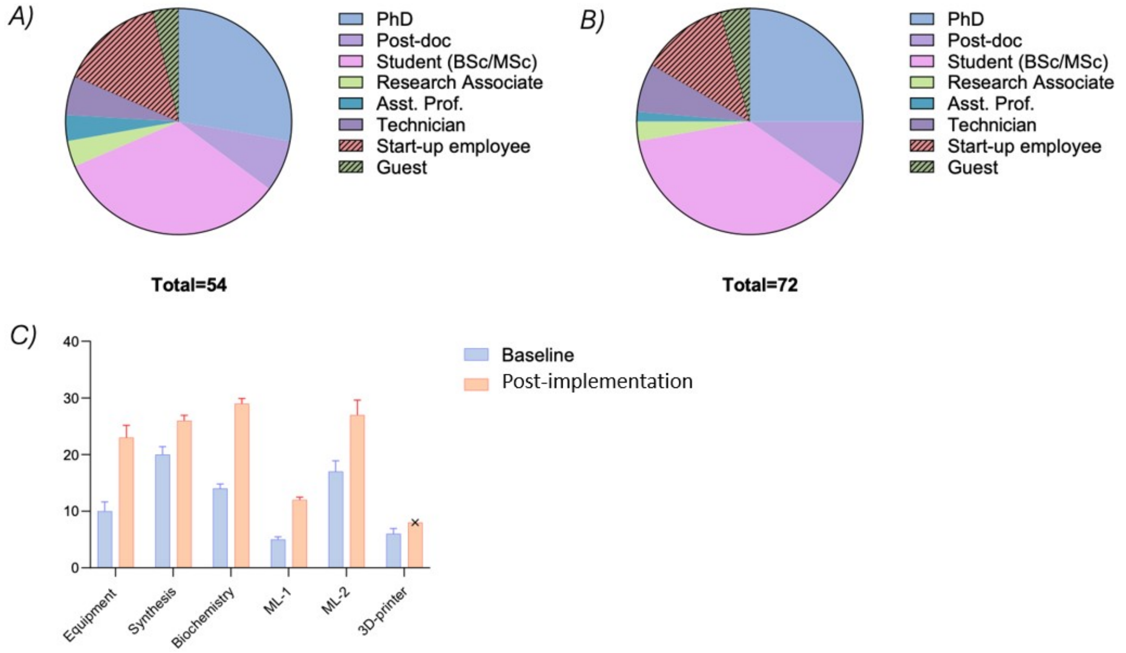

Figure S3. Lab-user population demographics in Px labs. **A-B)** Composition of lab users during **(A)** baseline and **(B)** post-implementation phases. Pattern-filled fractions denote external users (i.e., non-Px affiliated). **C)** Monthly average number of lab users during both phases, categorized by lab area. Data are presented as mean ± SD (N = 3). A cross symbol indicates no variation. Because many individuals worked across multiple areas, aggregated monthly counts exceed the total number of unique users.

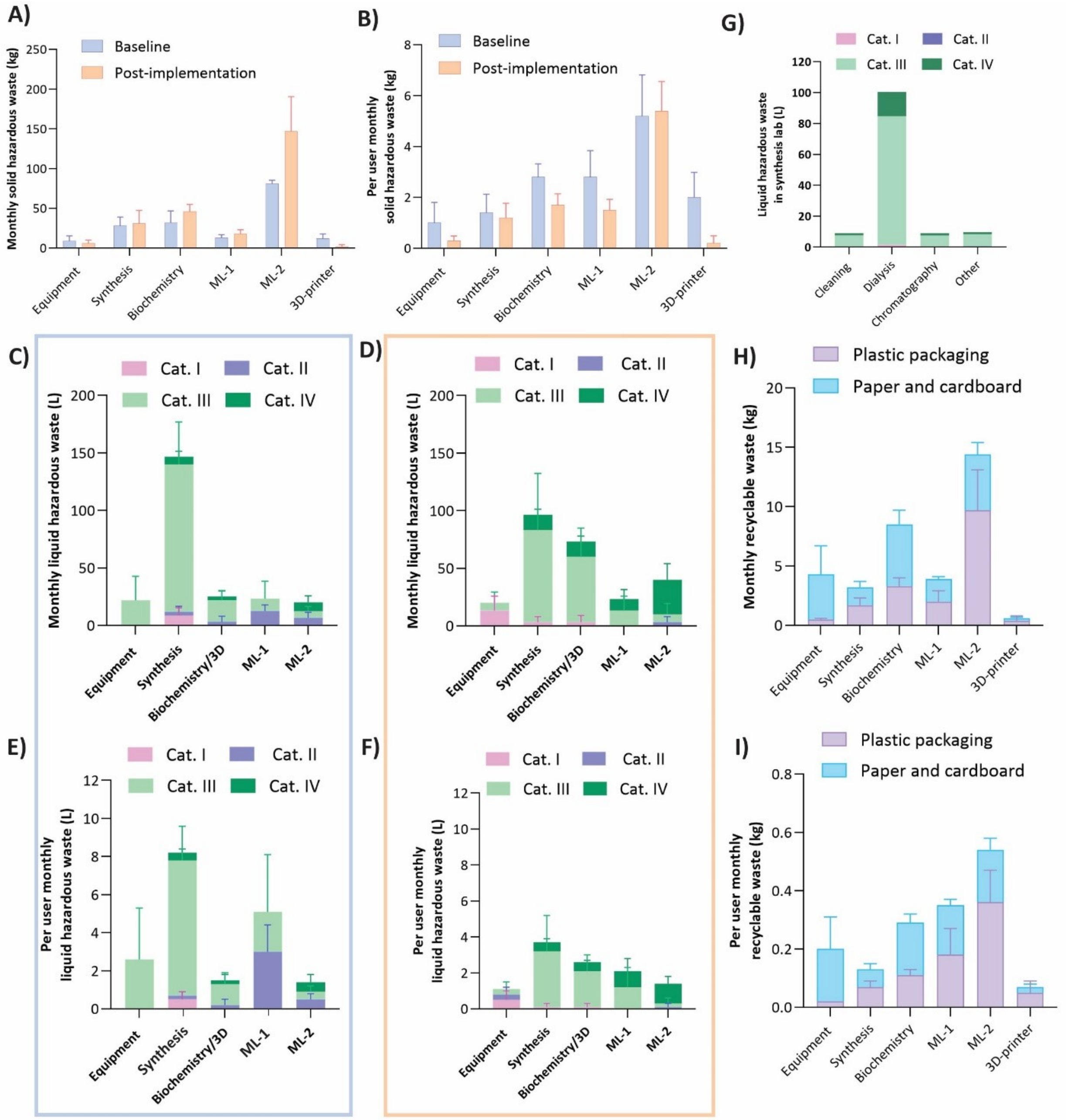

Figure S4. Quantification of hazardous and recyclable waste across Px lab areas. Lab hazard classifications are as follows: low biological hazard (ML-1), moderate biological hazard (ML-2), and chemical hazard (synthesis, biochemistry, 3D printing, and equipment labs). Within the chemical hazard category, lab areas are distinguished by experimental work type. Note: the 3D printing and biochemistry labs share a collection point for liquid hazardous waste disposal. **A)** Monthly production of solid hazardous waste pre- and post-intervention. **B)** Per-user monthly production of solid hazardous waste pre- and post-intervention. **C–D)** Monthly production of liquid hazardous waste pre- (C) and post-intervention (D). **E–F)** Per-user monthly production of liquid hazardous waste pre- (E) and post-intervention (F). **G)** Origin of liquid hazardous waste during the post-intervention phase in the synthesis lab. **H)** Monthly production of recyclables post-intervention. **I)** Per-user monthly production of recyclables post-intervention.
